# Optimization of an Industrial Medium and Culture Conditions for Probiotic *Weissella cibaria* JW15 Biomass Using the Plackett-Burman Design and Response Surface Methodology

**DOI:** 10.4014/jmb.2202.02020

**Published:** 2022-03-22

**Authors:** Hyung-Seok Yu, Na-Kyoung Lee, Won-Ju Kim, Do-Un Lee, Jong-Ha Kim, Hyun-Dong Paik

**Affiliations:** Department of Food Science and Biotechnology of Animal Resources, Konkuk University, Seoul 05029, Republic of Korea

**Keywords:** *Weissella cibaria*, probiotics, biomass production, plackett-burman design, response surface methodology

## Abstract

The objective of this study was to optimize industrial-grade media for improving the biomass production of *Weissella cibaria* JW15 (JW15) using a statistical approach. Eleven variables comprising three carbon sources (glucose, fructose, and sucrose), three nitrogen sources (protease peptone, yeast extract, and soy peptone), and five mineral sources (K_2_HPO_4_, potassium citrate, L-cysteine phosphate, MgSO_4_, and MnSO_4_) were screened by using the Plackett-Burman design. Consequently, glucose, sucrose, and soy peptone were used as significant variables in response surface methodology (RSM). The composition of the optimal medium (OM) was 22.35 g/l glucose, 15.57 g/l sucrose, and 10.05 g/l soy peptone, 2.0 g/l K_2_HPO_4_, 5.0 g/l sodium acetate, 0.1 g/l MgSO_4_·7H_2_O, 0.05 g/l MnSO_4_·H_2_O, and 1.0 g/l Tween 80. The OM significantly improved the biomass production of JW15 over an established commercial medium (MRS). After fermenting OM, the dry cell weight of JW15 was 4.89 g/l, which was comparable to the predicted value (4.77 g/l), and 1.67 times higher than that of the MRS medium (3.02 g/l). Correspondingly, JW15 showed a rapid and increased production of lactic and acetic acid in the OM. To perform a scale-up validation, batch fermentation was executed in a 5-l bioreactor at 37°C with or without a pH control at 6.0 ± 0.1. The biomass production of JW15 significantly improved (1.98 times higher) under the pH control, and the cost of OM was reduced by two-thirds compared to that in the MRS medium. In conclusion, OM may be utilized for mass producing JW15 for industrial use.

## Introduction

Intestinal microbiota, comprising a large number and high diversity of commensal microorganisms, are primarily associated with the health of their host owing to their modulatory effects on various biological functions such as homeostasis, digestion, and immunity [1, 2]. Probiotics are active microorganisms that provide the host with diverse health benefits, such as balancing intestinal compositions, preventing invasions of pathogens, and improving the immune system upon colonization of the intestinal microbiota [3, 4]. Probiotic microbes are commercially utilized in the food, pharmaceutical, and livestock farming industry, among industries grows annually, especially in European and Asian regions, with a forecast of 50 billion dollars in the following five years [5, 6]. As the importance of probiotics is consistently highlighted, research on their production at an industrial scale is required to satisfy growing demand [7].

Lactic acid bacteria (LAB) are typical probiotic microbes commonly found in fermented foods. Along with garnering attention for increasing overall well-being, LAB are garnering the interest for their ability to improve disorder-related conditions or prevent said disorders altogether [8, 9]. Probiotic LAB have known to exhibit therapeutic and prophylactic effect on various diseases, ranging from pathogen-induced diarrhea to degenerative disorders [2]. LAB contribute to improvement of intestinal microbiota by displaying antimicrobial, antioxidant, and immuno-modulatory activities [10, 11]. Moreover, LAB-derived bio-active compounds, such as γ-aminobutyric acid, short-chain fatty acid (SCFA)s, and exopolysaccharides, are have known to be involved in not only modulation of gut microbiota but also mitigation of colitis and neuronal disorders [12].

The most common commercial medium for LAB cultivation is de Man Rogosa Sharpe (MRS), however, this MRS medium cannot maximize the growth of LAB owing to their fastidious and complex nutrient requirements, depending on the strains. In addition, the cost of using the MRS medium for industrial purposes is comparatively high [13]. Therefore, an optimized medium must be developed to improve the biomass or metabolite production of LAB with respect to each strain and species. Statistical optimization methods, such as the Plackett-Burman (PB) design and response surface methodology (RSM), minimize the error in determining the effect of independent variables and facilitate deducing the optimal conditions by establishing the relationship between each parameter and predicted responses [14].

*Weissella cibaria* is a relatively recent member of various LAB species. *W. cibaria* is reportedly vital in kimchi fermentation as a dominant strain and exhibits various beneficial effects. In a previous study, *W. cibaria* JW15 (JW15) displayed exopolysaccharide production and antagonistic, antioxidant, and immunomodulatory functions [11, 15-17]. Furthermore, fermentation with JW15 increases the bioavailability of medicinal herbs in RAW 264.7 cells by attenuating the toxicity [18]. Although several studies have demonstrated the functional properties of *W. cibaria* strains, media optimization for improving biomass production of *W. cibaria* has not yet been reported. Therefore, the present study aimed to develop a culture medium for the industrial-scale production of JW15 with inexpensive industrial-grade materials. The media composition optimization was performed using the PB design and RSM.

## Materials and Methods

### Chemicals and Reagents

The MRS media and protease peptone were purchased from Difco Laboratories (USA). Industrial-grade materials, including glucose, fructose, sucrose, yeast extract, soy peptone, K_2_PO_4_, potassium citrate, L-cysteine phosphate, MgSO_4_, and MnSO_4_, were obtained from Lactomason, Co. Ltd. (Korea). Solvents for high-performance liquid chromatography (HPLC) were purchased from J.T. Baker (USA). All other chemicals were purchased from Sigma-Aldrich (USA).

### Bacterial Strains, Culture Conditions, and Inoculum Preparation

*W. cibaria* JW15 (KACC 91811P) was obtained as described in our previous study [16]. Stock culture was prepared in sterilized MRS broth containing 30% (v/v) of glycerol and stored at –80°C before use. To activate the JW15, stock culture was streaked on MRS agar plates and incubated for 24 h at 37°C. The strain was then sub-cultured in MRS broth (1%, v/v) in triplicate to prepare the seed culture.

Erlenmeyer flasks (250 ml) containing 200 ml of MRS broth were inoculated with 1% (v/v) seed culture and incubated for 24 h at 37°C. The bacterial cells were harvested through centrifugation (5,000 ×*g*, 10 min, 4°C) and washed twice with 0.85% sodium chloride aqueous solution (w/v). The collected pellets were re-suspended in each constructed culture medium to obtain an absorbance of 0.5 at 600 nm. The constructed culture media was inoculated with a 1% (v/v) inoculum ratio in all experiments as a standard.

### Experimental Design Using the Plackett-Burman Design

Nutrients that exhibited a significant effect on biomass formation in JW15 were screened using the PB design. The coded levels and actual values of each variable are presented in Table S1. The concentration range of the independent variables was determined based on the commercial medium (MRS). Eleven real variables (three carbon sources, three nitrogen sources, and five mineral sources) and four dummy variables were examined in 19 trials, including triplicates tests at the central point (Table 1). Each column and row represent an independent variable and a trial, respectively. Variables with a confidence level above 95% had a significant effect on the biomass production of JW15. The regression model for biomass production of JW15 is as follows:

$$Y=B_{0}+\sum B_{i}X_{i}$$

Where *Y* is the response (dry cell weight of JW15), and *B_0_*, *B_i_*, and *X_i_* is the constant, the respective linear coefficients, and the independent variables, respectively.

### Response Surface Methodology

Glucose, sucrose, and soy peptone were the most effective nutrients used in the biomass production of JW15. Thus, the basal media, comprised of 2.0 g/l dipotassium phosphate (K_2_HPO_4_), 5.0 g/l sodium acetate, 0.1 g/l magnesium sulfate heptahydrate (MgSO_4_·7H_2_O), 0.05 g/l manganese sulfate monohydrate (MnSO_4_·H_2_O), and 1.0 g/l Tween 80, was added to the constructed media in subsequent experiments. Response surface methodology (RSM) was employed to optimize the growth medium based on the central composite design (CCD). The CCD comprises the center point and star points (α, distance from the center point) as a fractional or full factorial design. The replication of experiment at the center point of the CCD is commonly conducted to improve the precision of the experimental model [19]. The number of experimental trials (N) and the value of α were calculated using Eqs. (1) and (2), respectively:

$$N=k^{2}+2k+C_{n}$$ (1)

$$\alpha ={\left(2^{k}\right)}^{1/4}$$ (2)

Where *k* is the number of independent variables and *C_n_* is the replication number at the center point. Following this, CCD was constructed three independent variables at five levels of concentration. The coded values and actual concentrations of each variable was presented in Table S2. The α value was 1.68 and 18 experimental trials were performed including replication in triplicate at the center point. The experimental data obtained from CCD was fitted to the second-order polynomial model as follows:

$$Y=B_{0}+\sum_{i=1}^{n}B_{i}X_{1}+\sum_{j\leq i}^{n}B_{ij}X_{i}X_{j}$$

Where *Y* is the predicted response (dry cell weight of JW15), *B_0_* is the constant term, *B_i_* is the linear coefficient, *B_ij_* is the quadratic coefficient, and *X_i_* and *X_j_* are the independent variables. After determining the optimal composition for the culture medium, biomass production of JW15 in an optimal medium (OM) was compared with that of a commercial medium (CM; MRS).

### Measurement of Biomass, Lactic Acid, and Acetic Acid

The biomass production of JW15 was determined by measuring the dry cell weights [20]. An aliquot (10 ml) of the culture broth was transferred to pre-weighed plastic conical tubes and centrifuged (5,000 ×*g*, 10 min, 4°C). The cell-free supernatant was separated to determine the concentration of organic acids. Pellets were washed thrice with ice-cold distilled water, dried at 80°C and left in a vacuum oven to obtain a constant weight.

The lactic and acetic acid production of JW15 was assessed using HPLC analysis, as described previously [16]. The obtained supernatant was passed through a 0.22-μm membrane filter and used for the HPLC analysis. Analysis was conducted using an Agilent 1100 apparatus (Agilent Technologies, USA), which comprises a column oven, auto-sampler, UV detector, and Aminex HPX-87H column (300 mm × 7.8 mm, 5 μm) (Bio-Rad, USA). The injection volume was 20 μl. Isocratic elution was performed using a 5 mM H_2_SO_4_ aqueous solution at 50°C. The flow rate was 0.6 ml/min. The chromatogram were detected at 210 nm using a UV detector. The concentration of each organic acid was evaluated from the external regression curve, which was constructed using standard mixtures at five concentrations.

### Effect of pH and Temperature

The effects of the initial pH and culture temperature were assessed to determine the optimal culture conditions for JW15. The initial pH was adjusted to 5.5, 6.0, and 6.5 with 3 M NaOH and 3 M HCl prior to sterilization, and the incubation temperature was maintained at 27, 32, 37, and 40°C. The optimal conditions were determined by comparing the viable cell numbers after 24 h of incubation.

### Scale-Up Fermentation

The biomass production of JW15 was determined in a 5-l bioreactor with OM to evaluate the scale-up fermentation. The working volume was 3.5 l and the inoculation ratio was 1% (v/v). The agitation speed and temperature were maintained at 200 rpm and 37°C, respectively. The pH was controlled at 6.0 ± 0.1 using 3 M hydrochloride (HCl) aqueous solution and 3 M sodium hydroxide (NaOH) aqueous solution during incubation. An aliquot (20 ml) of culture was collected to determine the cell viability, pH, and biomass during 24 h of fermentation. The obtained culture was appropriately diluted, spread on MRS agar plates, and incubated at 37°C for 24 h. Cell viability was then calculated. CM was used as a positive control.

### Statistical Analysis

The data are presented as the mean of the three values. The experiment was independently conducted in triplicate. Results were analyzed via one-way analysis of variance using SAS software (version 9.4; SAS Institute, USA). Differences between the two groups were determined with a one-tailed Student's t-test within IBM SPSS software (version 24.0; SPSS Inc., USA). Differences were considered significant when *p* < 0.05.

## Results and Discussion

### Plackett-Burman Design for Screening

The PB experimental design was used to screen the nutritional factors that had a significant effect on the biomass production of JW15. The concentrations for the independent variables were determined by considering the composition of CM. A total 15 variables comprising 11 real variables (3 carbon sources, 3 nitrogen sources, and 5 mineral sources) and 4 dummy variables with 19 experimental runs resulted in JW15 biomass production rates ranging from 0.93 to 2.98 g/l (Table 1). Table 2 presents the estimated effect of each variable on the biomass production of JW15. The analysis of variance showed that all variables except MnSO_4_ were statistically significant (*p* < 0.05). Based on the results of PB experimental design, glucose (0.72) had the most significant effect on JW15 biomass production, followed by the soy peptone (0.50) and sucrose (0.29). Therefore, glucose, sucrose, and soy peptone were the most significant variables for RSM.

### Response Surface Methodology for Media Optimization

Following the results of the PB design, the medium composition was optimized to maximize the JW15 biomass production using RSM with CCD. Considering the composition of CM, the concentrations of carbohydrate and nitrogen sources varied between 0 and 25.1 g/l and 0 and 12.7 g/l, respectively (Table S2). Table 3 represents the full experimental design matrix comprising a total of 20 runs along with the JW15 biomass production, which varied from 0.96 to 4.67 g/l. ANOVA was performed to verify the adequacy and significance of the second-order polynomial model. The coefficient of determination (R2 value) obtained for the response variable was 0.9740, indicating that the regression model adequately details the overall response. This regression model is highly significant, which is evident from the high F-value (33.30) and very low probability value (< 0.0001). In accordance with the results of the PB design, glucose was the most significant variable, followed by soy peptone and sucrose. In addition, the regression model was significantly affected by the linear effect. The accuracy of this regression model was verified by showing an insignificant lack of fit value (*p* = 0.2771), thereby demonstrating that the model appropriately expresses the result in the experimental domain even if the points are not involved in this regression [21]. Thus, the following model is proposed.

$$Y\;=\;2.4517\;+\;1.3500X_{1}\;+\;0.4102X_{3}\;+\;1.2207X_{6}\;+\;0.3238X_{1}{}^{2}\;-\;0.2706X_{3}{}^{2}\;-\;0.0437X_{6}{}^{2}\;+\;0.2033X_{1}X_{3}\;+\;0.3864X_{3}X_{6}\;+\;0.4397X_{6}X_{1}$$

**Fig. 1:**
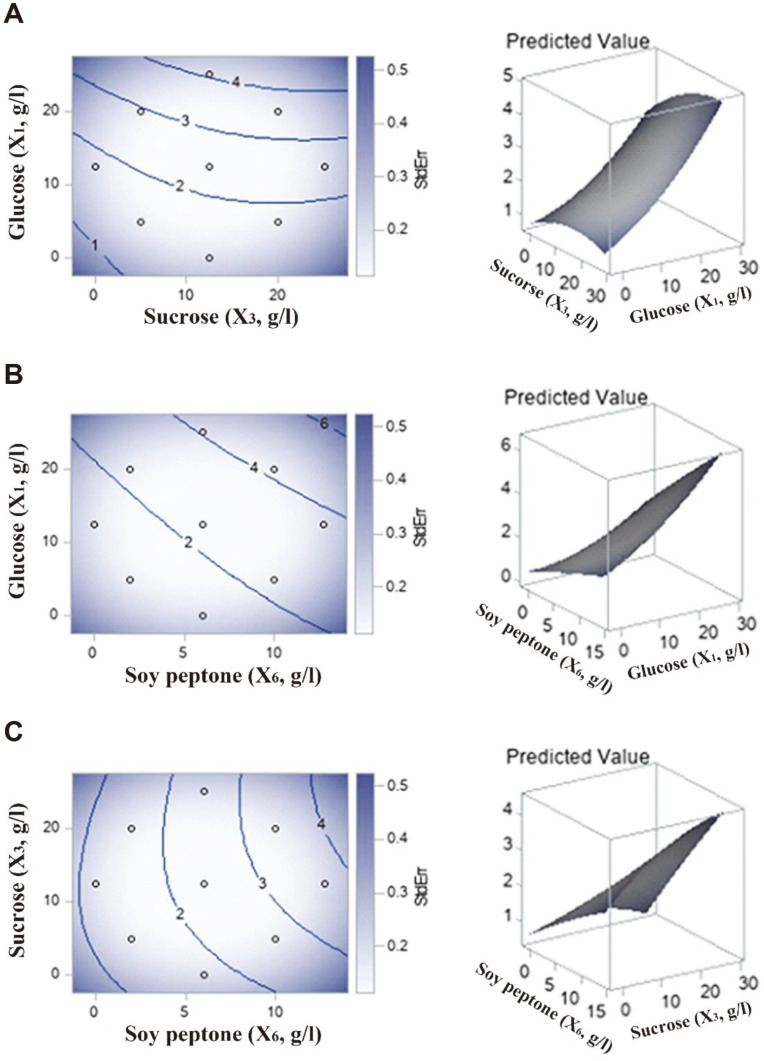
Contour plots and response surface plots for biomass production of *Weissella cibaria* JW15. **A**. Interaction between glucose (X_1_, g/l) and sucrose (X_3_, g/l) with soy peptone (X_6_, g/l) at zero level. **B**. Interaction between glucose (X_1_, g/l) and soy peptone (X_6_, g/l) with sucrose (X_3_, g/l) at zero level. **C**. Interaction between sucrose (X_3_, g/l) and soy peptone (X_6_, g/l) with glucose (X_1_, g/l) at zero level.

Contour plots and a three-dimensional response surface were drawn to reveal the interactions between the two variables and deduce the optimal concentration (Figs. 1A-1C). Owing to the linear effects of each variable, the predicted response variable showed a steady increment upon increasing the concentrations of variables. Therefore, the optimal concentrations of glucose (*X_1_*), sucrose (*X_3_*), and soy peptone (*X_6_*) were 1.000 of the coded radius level, at 22.35, 15.57, and 10.05 g/l, respectively. At the optimal concentration, the regression model predicted a maximized biomass production of JW15 of 4.79 g/l, with a 95% confidence interval ranging from 4.63 to 4.91 g/l.

**Fig. 2:**
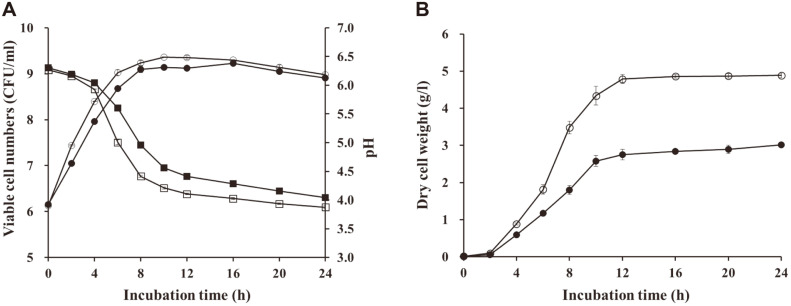
Time course of batch incubation of *Weissella cibaria* JW15 (JW15) in commercial medium (CM; MRS) and optimal medium (OM). JW15 was cultivated in an Erlenmeyer flask containing 200 ml of growth medium. **A**. Viable cell numbers (●, CM; ○, OM) and pH (■, CM; □, OM) were investigated during incubation. **B**. Biomass production of JW15 was estimated by measuring the dry cell weight (●, CM; ○, OM).

To validate the improvement in biomass production following statistical optimization, the time-course growth performance of JW15, with respect to changes in viable cell numbers, pH, and biomass in the OM and CM, was evaluated (Figs. 2A and 2B). Fermentation was conducted for 24 h in a 250-ml Erlenmeyer flask containing 200 ml of each medium. JW15 showed enhanced growth in OM compared to CM, demonstrating a rapid increase and decrease in viable cell numbers and pH, respectively (Fig. 2A). The maximum viable cell numbers in OM (9.36 ± 0.01 Log CFU/ml) and CM (9.23 ± 0.03 Log CFU/ml) were observed at the 12 h and 16 h of incubation, respectively. Consistently, the JW15 biomass production in OM was superior compared to that in CM (Fig. 2B). The JW15 biomass production dramatically increased between 2 and 12 h of incubation. Although the maximum biomass production was obtained at 24 h (4.89 ± 0.06) of incubation in both media, the shelving slopes were observed during biomass production after 12 h of incubation. Following 12 h of fermentation, the JW15 biomass production in OM was 1.74 times higher than that in CM, showing 4.79 ± 0.12 g/l and 2.75 ± 0.15 g/l of dry cell weight, respectively. Additionally, the regression model showed an acceptable agreement between the predicted and experimental values. Several studies have reported that an optimized medium following the statistical method enhances the biomass and/or metabolites production of various LAB, such as *Lactobacillus plantarum*, *Lactobacillus reuteri*, *Lactobacillus casei*, and *Leuconostoc mesenteroides* [3, 6, 20, 22, 23]. Similar to our results, a statistically optimized medium containing soytone significantly improved the biomass production of *L. plantarum* 200655 compared to CM [3].

### Organic Acid Production

LAB produce various organic acids by metabolizing diverse carbohydrates. Among the organic acids, lactic acid is a major metabolite and a crucial parameter in estimating the growth of LAB [24]. The lactic and acetic acid formation of JW15 was examined during fermentation. Table 5 represents the time course of lactic and acetic acid production in the OM and CM, respectively. In accordance with the improved growth rate and JW15 biomass production (Figs. 2A and 2B), JW15 showed significantly (*p* < 0.05) faster and higher lactic and acetic acid production in OM than in CM. However, the concentration of lactic acid was lower, but that of acetic acid was higher in OM than in CM after 24 h of incubation. Following glucose depletion, LAB will convert lactic acid into acetic acid under aerobic condition. Accordingly, long-term fermentation of LAB results in the accumulation of acetic acid as a substitute for lactic acid and subsequently induces cell pH homeostasis [20, 24]. Overall, OM better improved the growth of JW15 than CM.

### Comparison of Biomass Production in 5-l Bioreactor

Microbial growth is affected by media composition as well as physicochemical properties. Thus, the effects of pH and incubation temperature on the growth of JW15 were evaluated prior to performing the scale-up fermentation. The optimal pH and temperature of most LAB for biomass production reportedly range from 5.0 to 7.0 and from 25 to 40°C, respectively [20, 22, 25]. Optimal culture conditions for the growth of JW15 were investigated based on the results of the PB design and RSM. Fig. S1A and S1B represent the growth rate of JW15 with respect to pH and temperature, respectively. JW15 showed the fastest increase in viable cell numbers at pH 6.0 (Fig. S1A) and 37°C (Fig. S1B), respectively. These results were in accordance with a previous study describing the optimal pH and temperature for the highest specific growth rate of *W. cibaria* DBPZ1006 following dynamic modeling [26]. There was a slight difference in the optimum pH; however, several studies have reported that even when using the same strain, optimum pH, and temperature for growth, biomass production may differ depending on the origin of isolation [3, 11]. Therefore, the optimum pH and temperature were determined at pH 6.0 and 37°C in the subsequent scale-up fermentation.

**Fig. 3:**
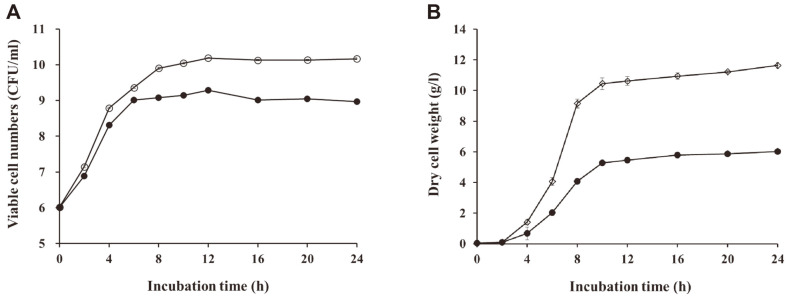
Time course of batch incubation of *Weissella cibaria* JW15 (JW15) optimal medium (OM) with or without adjustment of pH. JW15 was cultivated in a 5-l bioreactor containing 3.5 l of OM without or with pH control at 6.0 ± 0.1. **A**. Viable cell numbers were determined during incubation (●, without pH control; ○, with pH control). **B**. Biomass production of JW15 was evaluated by measuring the dry cell weight (●, without pH control; ○, with pH control).

Batch fermentation was conducted for 24 h in a 5-l bioreactor containing 3.5 l of OM at 37°C and 200 rpm of agitation with or without a controlled pH at 6.0 ± 0.1. Fig. 3 shows the time-course viable cell numbers and biomass production of JW15 during fermentation. The growth rate of JW15 significantly (*p* < 0.05) improved upon controlling the pH at 6.0 ± 0.1 that in the pH control groups (Fig. 3A). A quick increase in viable cell numbers was observed between 0 and 8 h of incubation under pH control. In addition, the highest viable cell numbers were observed after 12 h of incubation in pH-controlled groups (10.19 ± 0.02 Log CFU/ml) and pH-uncontrolled group (9.15 ± 0.02 Log CFU/ml). pH controls consistently and significantly enhanced the biomass production of JW15 (Fig. 3B) (*p* < 0.05). Accordingly, the JW15 biomass production dramatically increased between 4 and 10 h of fermentation. The maximized biomass production was obtained at 24 h of fermentation; however, the increase in biomass production displayed a gentle slope between 10 and 24 h of incubation. Following 10 h of incubation, the JW15 production in pH-controlled groups (10.44 ± 0.38 g/l) was 1.98 times higher than those in pH-uncontrolled groups (5.28 ± 0.13 g/l). These findings are supported by several previous studies that have improved biomass production upon using a pH control; for example, control of pH facilitated and prevented the accumulation of residual carbohydrates, which led to the continuous growth of microorganisms [3, 22, 27, 28].

Collectively, the biomass production of JW15 may be improved in an OM comprising 22.35 g/l glucose, 15.57 g/l sucrose, and 10.05 g/l soy peptone, 2.0 g/l K_2_HPO_4_, 5.0 g/l sodium acetate, 0.1 g/l MgSO_4_·7H_2_O, 0.05 g/l MnSO_4_·H_2_O, and 1.0 g/l Tween 80 under culture conditions of pH 6.0, 37°C, and an agitation speed of 200 rpm. Additionally, the cost of optimal media is two-thirds that of CM. In conclusion, optimized media composition and culture conditions using statistical methods may be utilized for enhancing biomass production of JW15 at an industrial-scale.

## Supplemental Materials

Supplementary data for this paper are available on-line only at http://jmb.or.kr.

## Figures and Tables

**Table 1:** Experimental matrix of the Plackett-Burman design for biomass production of *Weissella cibaria* JW15.

Trial no.	Variables^a^/levels^b^															Dry cell weight (g/l)^c^

	X_1_	X_2_	X_3_	X_4_	X_5_	X_6_	X_7_	X_8_	X_9_	X_10_	X_11_	D_1_	D_2_	D_3_	D_4_	
1	–1	–1	–1	–1	+1	+1	+1	+1	+1	+1	–1	–1	–1	–1	+1	1.32
2	–1	–1	–1	–1	–1	–1	–1	+1	+1	+1	+1	+1	+1	–1	–1	0.93
3	–1	+1	–1	–1	–1	+1	+1	–1	–1	+1	+1	+1	–1	+1	–1	1.29
4	+1	+1	–1	–1	+1	–1	–1	+1	–1	+1	–1	–1	+1	+1	+1	1.23
5	–1	–1	+1	–1	+1	–1	+1	–1	+1	–1	+1	–1	+1	+1	–1	1.44
6	+1	–1	+1	–1	–1	+1	–1	–1	+1	–1	–1	+1	–1	+1	+1	2.98
7	–1	+1	+1	–1	–1	–1	+1	+1	–1	–1	–1	+1	+1	–1	+1	1.37
8	+1	+1	+1	–1	+1	+1	–1	+1	–1	–1	+1	–1	–1	–1	–1	2.26
9	–1	–1	–1	+1	+1	+1	–1	+1	–1	–1	–1	+1	+1	+1	–1	2.05
10	+1	–1	–1	+1	–1	–1	+1	+1	–1	–1	+1	–1	–1	+1	+1	1.63
11	–1	+1	–1	+1	–1	+1	–1	–1	+1	–1	+1	–1	+1	–1	+1	1.60
12	+1	+1	–1	+1	+1	–1	+1	–1	+1	–1	–1	+1	–1	–1	–1	2.50
13	–1	–1	+1	+1	+1	–1	–1	–1	–1	+1	+1	+1	–1	–1	+1	1.47
14	+1	–1	+1	+1	–1	+1	+1	–1	–1	+1	–1	–1	+1	–1	–1	1.70
15	–1	+1	+1	+1	–1	–1	–1	+1	+1	+1	–1	–1	–1	+1	–1	1.15
16	+1	+1	+1	+1	+1	+1	+1	+1	+1	+1	+1	+1	+1	+1	+1	2.53
17	0	0	0	0	0	0	0	0	0	0	0	0	0	0	0	1.76
18	0	0	0	0	0	0	0	0	0	0	0	0	0	0	0	1.75
19	0	0	0	0	0	0	0	0	0	0	0	0	0	0	0	1.77

^a^X_1_, glucose; X_2_, fructose; X_3_, sucrose; X_4_, protease peptone; X_5_, yeast extract; X_6_, soy peptone; X_7_, K_2_HPO_4_; X_8_, potassium citrate; X_9_, L-cysteine phosphate; X_10_, MgSO_4_; X_11_, MnSO_4_; D_1_, D_2_, D_3_, and D_4_ are dummy variables.

^b^(+1), highest concentration of variable; (–1), lowest concentration of variable; (0), central concentration of variable.

^c^The dry cell weight was measured after 24 h of incubation. Data are presented as the mean of independent experiments performed in triplicate.

**Table 2:** Estimated effects of variables for biomass production from the Plackett-Burman design results.

Independent variables (g/l)	Estimated effect	*P*-value
Glucose (X_1_)	0.72	0.000
Fructose (X_2_)	0.05	0.014
Sucrose (X_3_)	0.29	0.000
Protease peptone (X_4_)	0.23	0.000
Yeast extract (X_5_)	0.27	0.000
Soy peptone (X_6_)	0.50	0.000
K_2_HPO_4_ (X_7_)	0.02	0.000
Potassium citrate (X_8_)	–0.24	0.000
L-Cysteine phosphate (X_9_)	0.18	0.000
MgSO_4_ (X_10_)	–0.13	0.000
MnSO_4_ (X_11_)	–0.15	0.299

**Table 3:** Experimental matrix of central composite design for biomass production of *Weissella cibaria* JW15.

Run	Variables (g/l)			Dry cell weight (g/l)^a^

	Glucose	Sucrose	Soy peptone	
1	5	5	2	0.96
2	20	5	2	1.99
3	5	20	2	1.26
4	20	20	2	2.43
5	5	5	10	1.96
6	20	5	10	3.50
7	5	20	10	2.69
8	20	20	10	4.67
9	0	12.5	6	1.12
10	25.1	12.5	12.7	4.12
11	12.5	0	6	1.87
12	12.5	25.1	6	2.18
13	12.5	12.5	0	1.1
14	12.5	12.5	12.7	3.52
15	12.5	12.5	6	2.56
16	12.5	12.5	6	2.28
17	12.5	12.5	6	2.18
18	12.5	12.5	6	2.52

^a^The dry cell weight was measured after 24 h of incubation. Data are presented as the mean of independent experiments performed in triplicate.

**Table 4:** Analysis of variance for the quadratic polynomial model of the biomass production of *Weissella cibaria* JW15.

Regression	DF	Sum of squares	R-square	*F*-value	Pr > F
Linear	3	16.891383	0.9306	95.44	< 0.0001
Quadratic	3	0.360026	0.0198	2.03	0.1877
Cross product	3	0.428050	0.0236	2.42	0.1414
Total model	9	17.679459	0.9740	33.30	< 0.0001

Residual	DF	Sum of squares	Mean square	*F*-value	Pr > F

Lack of fit	5	0.370069	0.074014	2.18	0.2771
Pure error	3	0.101900	0.33967		
Total error	8	0.471969	0.058996		

**Table 5:** Organic acid production of *Weissella cibaria* JW15 during batch fermentation.

Organic acid	Media	Concentration (g/kg)					

		0 h	2 h	4 h	6 h	12 h	24 h
Lactic acid	CM^A^	N.D.^C^	0.20 ± 0.01^a^	1.38 ± 0.01^a^	2.87 ± 0.07^a^	6.26 ± 0.06^a^	8.89 ± 0.05^b^
	OM^B^	N.D.	0.23 ± 0.01^b^	1.78 ± 0.04^b^	3.60 ± 0.03^b^	6.71 ± 0.09^b^	8.40 ± 0.21^a^
Acetic acid	CM	N.D.	0.05 ± 0.04^a^	0.14 ± 0.03^a^	0.28 ± 0.03^a^	0.71 ± 0.04^a^	0.95 ± 0.06^a^
	OM	N.D.	0.09 ± 0.00^b^	0.18 ± 0.03^b^	0.50 ± 0.03^b^	1.14 ± 0.03^b^	1.45 ± 0.02^b^

^A^CM, commercial media (MRS); ^B^OM, optimized media; ^C^N.D., not detected.

^a–b^Superscripts in the same row indicate statistical significance based on Student's *t*-test.

Data are presented as the mean ± standard deviation of independent experiments performed in triplicate.
