# Supplementary material for: Optimization of an Industrial Medium and Culture Conditions for Probiotic Weissella cibaria JW15 Biomass Using the Plackett-Burman Design and Response Surface Methodology
Source: J Microbiol Biotechnol. 2022 Mar 22;32(5):630–7. doi: 10.4014/jmb.2202.02020 (PMC9628880; doi:10.4014/jmb.2202.02020)
Supplement: Supplementary file 1 [file jmb-32-5-630-supple.pdf]

J. Microbiol. Biotechnol.
https://doi.org/10.4014/jmb.2202.02020

Supplementary Figures and Tables

Table S1. Coded levels and real values of factors for the Plackett-Burman design.

Independent variables (g/l)	Coded levels		
	–1	0	+1
Glucose (X_1)	5	10	15
Fructose (X_2)	5	10	15
Sucrose (X_3)	5	10	15
Protease peptone (X_4)	2	6	10
Yeast extract (X_5)	2	6	10
Soy peptone (X_6)	2	6	10
K_2HPO_4 (X_7)	1	2	3
Potassium citrate (X_8)	1	2	3
L-Cysteine phosphate (X_9)	0.2	0.5	0.8
$MgSO_4$ (X_{10})	0.05	0.1	0.15
$MnSO_4$ (X_{11})	0.002	0.005	0.008

Table S2. Coded levels and real values of factors for the central composite design.

Independent variables (g/l)	Coded levels				
	$-\alpha$	-1	0^a	$+1$	$+\alpha^b$
Glucose (X_1)	0	5	12.5	20	25.1
Sucrose (X_3)	0	5	12.5	20	25.1
Soy peptone (X_6)	0	2	6	10	12.7

[a] center point of central composite design, [b] star point of central composite design.

Fig. S1.

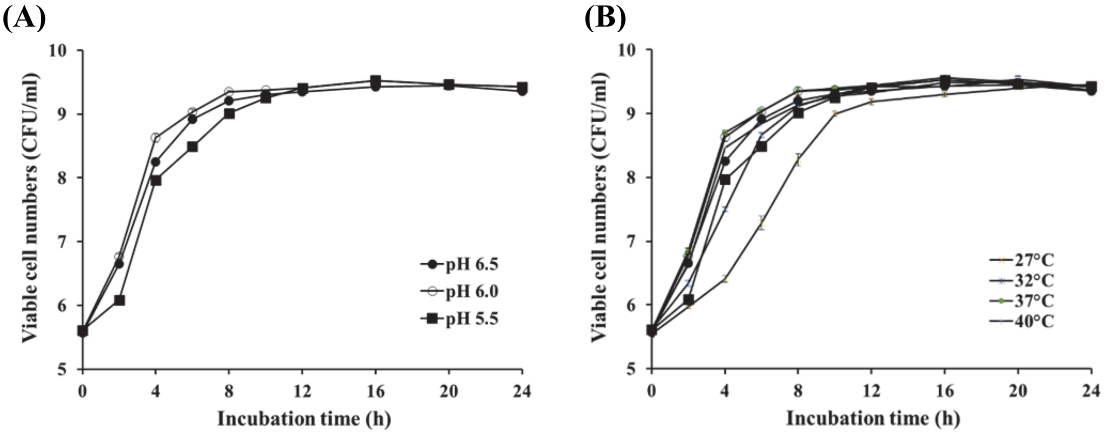

Figure S1. Effect of (A) pH and (B) temperature on the growth of *Weissella cibaria* JW15 in an optimal medium.
